# Metabolomic changes in crown of alfalfa (*Medicago sativa* L.) during de-acclimation

**DOI:** 10.1038/s41598-022-19388-x

**Published:** 2022-09-02

**Authors:** Zhensong Li, Feng He, Zongyong Tong, Xianglin Li, Qingchuan Yang, David B. Hannaway

**Affiliations:** 1 Institute of Animal Science, Chinese Academy of Agricultural Sciences, No.2, Yuanmingyuan West Road, Haidian District, Beijing, 100193 China; 2 Department of Crop & Soil Science, Oregon State University, Corvallis, USA

**Keywords:** Plant stress responses, Secondary metabolism

## Abstract

Alfalfa is a high-quality forage legume species that is widely cultivated at high latitudes worldwide. However, a decrease in cold tolerance in early spring seriously affects regrowth and persistence of alfalfa. There has been limited research on the metabolomic changes that occur during de-acclimation. In this study, a liquid chromatography–mass spectrometry system was used to compare the metabolites in two alfalfa cultivars during a simulated overwintering treatment. In four pairwise comparisons, 367 differential metabolites were identified, of which 31 were annotated according to the Kyoto Encyclopedia of Genes and Genomes (KEGG) database. Many of these metabolites were peptides, carbohydrates, and lipids. At the subclass level, 17 major pathways were revealed to be significantly enriched (*P* < 0.05). The main differential metabolites included amino acids, peptides and analogs, carbohydrates, and glycerol phosphocholines. A metabolomic analysis showed that the up-regulation of unsaturated fatty acids and amino acids as well as the enhancement of the related metabolic pathways might be an effective strategy for increasing alfalfa cold tolerance. Furthermore, glycerophospholipid metabolism affects alfalfa cold tolerance in early spring. Study results provide new insights about the changes in alfalfa metabolites that occur during de-acclimation, with potential implications for the selection and breeding of cold-tolerant cultivars.

## Introduction

In temperate climates, seasonal changes of alfalfa (*Medicago sativa* L.) include cold acclimation in autumn, dormancy in winter, and de-acclimation and regrowth in spring. For optimal survival, alfalfa acquires an adequate level of freezing tolerance, which is maintained for several months, and then de-acclimates after early spring freeze–thaw cycles. In northern China, because of the extended periods of sub-zero air and soil temperatures, only cold tolerant cultivars can be grown. These cultivars can tolerate extremely low temperatures during winter, but they can also adapt to temperature fluctuations in spring^1,2^.

A series of physiological, biochemical, and molecular changes induced by photoperiod and temperature maximize the freezing tolerance of plants during acclimation^3–5^. Earlier research indicated that the hardiness of perennial ryegrass (*Lolium perenne* L.)^6^ and asparagus (*Asparagus officinalis* L.)^7,8^ is negatively associated with increasing water contents. A decrease in the water content can lower the freezing point of cells, whereas an increase in the water content may result in mechanical damages due to extracellular freezing as well as an increase in the ice propagation rate in tissues^7–9^. Furthermore, the “energy-competitive hypothesis” states that the allocation of substances during acclimation will affect the freezing tolerance of plants, with a decrease in metabolizable energy consumption and an increase in energy production conducive to low-temperature stress tolerance when the water content is low^5^. The soluble sugar content in alfalfa root crowns reportedly increases several-fold in a few hours under low-temperature conditions^10^. Additionally, the superior winter hardiness of some cultivars is related to the early initiation of the accumulation of soluble sugars in autumn and high sugar levels^11,12^. Previous studies revealed that the differences between non-hardy and winter-hardy alfalfa cultivars are related to the capacity of plants to accumulate starch, sucrose, glucose, stachyose, raffinose, maltose, and fructose^8,10–13^. These soluble sugars prevent plants from being damaged by an exposure to low temperatures by maintaining the cell membrane integrity^8^, transmitting cold signals^14^, and regulating the expression of light-induced genes^3^ during acclimation. The plasma membrane is the primary site of freezing damage, the extent of which is closely associated with plasma membrane characteristics^15,16^. Thus, the plasma membrane lipid composition is an important factor affecting freezing tolerance. The molecular species composition of plasma membrane lipids has been investigated in Arabidopsis (*Arabidopsis thaliana* L.)^15^, winter wheat (*Triticum aestivum* L.)^17^, and alfalfa^13^, which differ in winter hardiness. The results of these investigations indicated that an increase in the proportion of unsaturated phospholipids leads to a proportional increase in the acquired cold hardiness. An increase in the abundance of certain lipid classes enhances the cryostability of the plasma membrane, which ultimately leads to the acquisition of freezing tolerance^13,15,18,19^.

The loss of cold hardiness due to de-acclimation has attracted the attention of researchers because cold-injury events are relatively frequent in early spring^20^. The term ‘de-acclimation’ is defined as a process in which the hardiness originally acquired through an earlier acclimation process decreases in response to environmental stimuli (i.e., warm temperatures and long days), phenological changes, and the reactivation of growth^5^. Plants in the de-acclimation period may be damaged by freeze–thaw cycles because: (1) compared with acclimation, de-acclimation may be a relatively less energy-intensive process, and cold hardiness decreases faster than the rate of environmental change^21,22^ and (2) increased metabolism results in fewer carbohydrates available for protecting plants from the detrimental effects of temperature fluctuations^23^. There is some controversy regarding the relationship between the water content and cold hardiness. For example, the water content of winter wheat and rye (*Secale cereale* L.) crowns increases throughout the de-acclimation period^24,25^, whereas the opposite change has been reported for *Rhododendron japonicum* and *Prunus avium*^26,27^. Soluble sugar levels decrease during de-acclimation as carbon losses exceed carbon gains at slightly elevated winter temperatures^5^. This is because low temperatures decrease mitochondrial respiration less than photosynthesis^23,28^. Previous studies demonstrated that the rate of the decrease in sugar levels increases significantly after an exposure to a short period of warm conditions, with a linear correlation between soluble sugar levels and hardiness^29,30^. The raffinose and stachyose contents in alfalfa^1^, the fructose, glucose, and sucrose contents in reed canary grass (*Phalaris arundinacea* L.)^31^ and cabbage (*Brassica oleracea* L.)^32^, and the total soluble sugar content in forsythia (*Forsythia suspensa* L.)^33^ decrease during de-acclimation. Moreover, Iivonen^34^ and Yoshida^35^ confirmed that de-acclimation is linked to the change in lipid metabolism in plants and cold hardiness is associated with decreases in several ratios (i.e., unsaturated to saturated phospholipids, phospholipids to proteins, and phospholipids to sterols). Related studies on alfalfa, Arabidopsis, and seashore paspalum (*Paspalum vaginatum* Sw.) indicated that an increase in glycerophospholipid, eicosanoid, and linoleic acid contents can increase cold hardiness^13,19,36,37^.

Although various aspects of acclimation in plants have been thoroughly investigated, the metabolic activities during de-acclimation remain relatively uncharacterized. In this study, we analyzed the changes in metabolite contents in differentially cold-hardy cultivars during de-acclimation and their contribution to cold tolerance.

## Materials and methods

### Plant materials

This study was conducted at the Institute of Animal Science, Chinese Academy of Agricultural Sciences (Beijing, China) from August 2020 to February 2021. The two alfalfa cultivars selected for this study were ‘WL440’ (fall dormancy score: approximately 6) and ‘ZhaoDong’ (ZD; fall dormancy score: approximately 2), which are commonly grown in northern China. These fall dormancy scores are representative of the alfalfa cultivars grown in the region. The WL440 seeds were provided by Beijing Zhengdao Seed Industry Co., Ltd. (Beijing, China), whereas the ZD seeds were obtained from Gansu Agricultural University (Lanzhou, China). Seeds were disinfected in sodium hypochlorite (1% NaClO) for 30 min and then washed five times with deionized water. Seeds similar in size were selected and germinated for 48 h in a Petri dish (diameter of 9 cm) at 25 °C. Eight germinated seeds were transferred into a polyvinyl chloride (PVC) pipe (inner diameter of 11 cm and length of 25 cm). Each PVC pipe was filled with 600 g sterilized dry mixture of sandy soil and nutrient soil (4:1, v/v). The dry mixture consisted of peat moss and lime, with 1.6% total nitrogen, 0.1% P_2_O_5_, 0.2% K_2_O (N:P:K = 14:10:18), 91% organic matter, conductivity of 0.9 dS m^−1^. The water-holding capacity of the mixture was 38.35%. Four plants that were about 20 cm tall were retained in each PVC pipe 2 weeks later. They were then allowed to grow for an additional 5 months, during which mowing was performed on September 30, November 10, and December 20, 2020, with 10 cm of stubble left after each mowing. The pipes were weighed every 4 days to check that the soil moisture content was maintained between 60 and 65% of water-holding capacity. Weeds and pests were removed regularly. The use of plants in the present study complies with international, national and/or institutional guidelines.

### Experimental design and sampling

**Figure 1:**
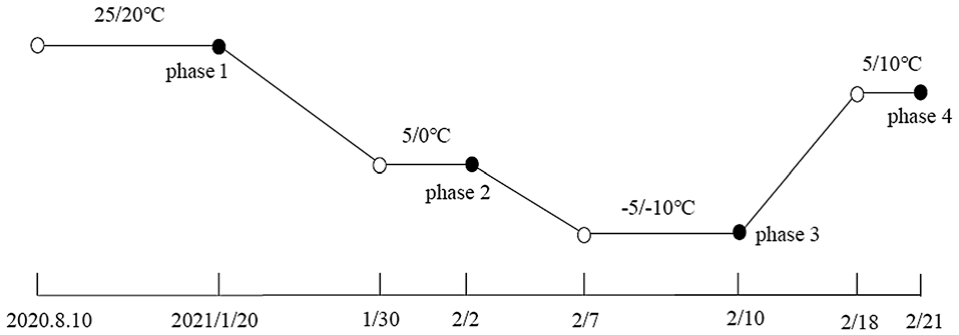
Schematic diagram of sampling in four test phases. Black dots for phases 1 to 4 represent sampling times in each phase.

The study was completed using a randomized complete block design involving two alfalfa cultivars and four experimental phases (Fig. 1) with six replicates for a total of 48 PVC pipes. From August 10, 2020 to January 20, 2021, all PVC pipes were incubated in an RXZ-500D artificial climate chamber (Ningbo Jiangnan Instrument Factory, Jiangsu, China) under the following conditions: 14 h day (25 °C)/10 h night (20 °C); 350 µmol·m^−2^·s^−1^ (photosynthetic photon flux density); 65% relative humidity. Phase 1 sampling occurred on January 20, 2021. The remaining materials were transferred to the LRH-200-GD low-temperature illumination incubator (Taihong Medical Instruments, Guangdong, China) for the subsequent analyses. The initial incubation conditions were as follows: 14 h day (25 °C)/10 h night (20 °C); 350 µmol·m^−2^·s^−1^ (photosynthetic photon flux density). The temperature was decreased to 5 °C (day)/0 °C (night) at a rate of 2 °C·d^−1^ and the light intensity was decreased to 150 µmol·m^−2^·s^−1^ at a rate of 20 µmol·m^−2^·s^−1^·d^−1^ to simulate the cold acclimation environment for alfalfa in autumn. Phase 2 sampling was performed after an additional 72 h of cold acclimation. The temperature was decreased further to − 5 °C (day)/ − 10 °C (night). Phase 3 sampling was conducted after a 72 h incubation. The temperature was then increased to 10 °C (day)/5 °C (night) at a rate of 2 °C·d^−1^. Phase 4 sampling was performed on February 21, 2021. The aboveground and belowground plant parts were separated and the root was carefully rinsed by hand with distilled water. The root crown (about 5 cm belowground) was divided into two parts. One part was used for measuring electrical conductivity, whereas the other part was stored at − 80 °C for the metabolomic analysis. The remainder of the root was measured to calculate the biomass. The aboveground and belowground parts were weighed after being placed in an oven at 65 °C for 48 h. Dry weights were recorded as the aboveground and belowground biomass.

### Determination of the semi-lethal temperature

The temperature at which the relative permeability of intracellular ions reaches 50% (LT_50_) was used to represent the cold hardiness of alfalfa^38^. The root-crown portion of plants were sliced into nine pieces (2–3 mm each) and placed in separate 2 mL centrifuge tubes. Tubes were kept at 4 °C prior to testing. The subsequent freezing test was conducted using the ZX-5C constant-temperature circulator (Zhixin Instrument, Shanghai, China). Sample tubes were incubated in alcohol for 1.5 h at nine decreasing temperatures. The alcohol temperatures differed because of the changes in LT_50_ among the four phases. For phase 1 samples, the nine temperatures were: 4, 2, 0, − 2, − 4, − 6, − 8, − 10, and − 12 °C. For phase 2 and 4 samples, the nine temperatures were: 0, − 2, − 4, − 6, − 8, − 10, − 12, − 14, and − 16 °C. For phase 3 samples, the nine temperatures were: − 4, − 6, − 8, − 10, − 12, − 14, − 16, − 18, and − 20 °C. After a 1.5 h incubation at one temperature, one tube was transferred to storage at that temperature. This was repeated until the incubations at all nine temperatures were completed. Subsequently, all samples were incubated at 4 °C for 2 h and then transferred to 15 mL centrifuge tubes. After adding 5 mL of deionized water, the tubes were shaken (120 rpm) on a HZQ-A gyratory platform shaker (Hengrui Instrument and Equipment, Changzhou, China) for 12 h at 25 °C. Electrical conductivity (i.e., EL_1_) was measured using a FE38 conductivity meter (Mettler, Shanghai, China). Samples were autoclaved at 121 °C for 30 min and electrical conductivity was remeasured (i.e., EL_2_). Electrical conductivity of the deionized water was also measured (i.e., EL). Relative electrolyte leakage was calculated according to Eq. (1) and the semi-lethal temperature was calculated using logistic Eq. (2), in which x is the freezing temperature, y is the relative electrolyte leakage, and A, B, and k are constants:

1 $$ {\text{Relative}}\;{\text{electrolyte}}\;{\text{leakage}}\;(\% ) = \left( {{\text{EL}}_{1} - {\text{EL}}} \right)/\left( {{\text{EL}}_{2} - {\text{EL}}} \right) \times 100 $$

2 $$ {\text{y}}\;(\% ) = {\text{A}}/\left( {1 + {\text{B}} \times {\text{e}}^{{ - {\text{kx}}}} } \right) \times 100 $$

### Metabolite extraction

To determine the changes in metabolite contents during de-acclimation, phase 3 and 4 samples (i.e., de-acclimation stages) were analyzed. Phase 3 and 4 samples of ZD and WL440 were designated ZD_1, ZD_2 and WL440_1, WL440_2. Frozen crown samples were ground to a fine powder in liquid nitrogen. 50 mg was transferred to a 2 mL centrifuge tube and a grinding ball (diameter of 6 mm) was added. 400 μL extraction solution was added composed of methanol and water (4:1, v/v) and 0.02 mg mL^−1^ L-2-chlorophenylalanine. Samples were ground for 6 min at − 10 °C using a grinder (50 Hz). An ultrasonic extraction (40 kHz) was performed for 30 min at 5 °C and samples were transferred to a centrifuge tube and incubated at − 20 °C for 30 min. The mixture was centrifuged at 13,000 g at 4 °C for 15 min. 200 μL supernatant was then added to an LC-MS sample vial. 20 μL supernatant aliquots from each sample were mixed to prepare quality control samples. To monitor the stability and repeatability of the instrumental analysis, the quality control samples (inserted after every eight samples) were analyzed along with an equal volume of each sample.

### Liquid chromatography-mass spectrometry (LC-MS) analyses

The LC-MS analyses were performed using ultra-high performance liquid chromatography (UHPLC)-Q Extactive HF-X system (Thermo Fisher Scientific, Waltham, MA, USA). Chromatographic separation was completed using the ACQUITY UPLC HSS T3 column (100 mm × 2.1 mm i.d., 1.8 μm; Waters, Milford, USA). Mobile phase A was 95% water and 5% acetonitrile (containing 0.1% formic acid). Mobile phase B was 47.5% acetonitrile, 47.5% isopropanol, and 5% water (containing 0.1% formic acid). The injection volume was 2 μL. The column temperature was 40 °C. Details regarding the mobile phase elution gradient are provided in Table S1. Samples were ionized (via electrospray) and mass spectrometry signals were collected in positive and negative ion scanning modes. The specific parameters are provided in Table S1.

### Bioinformatics and data analyses

Data were analyzed using the metabolome data processing software Progenesis QI (Waters Corporation) (i.e., for baseline filtration, peak identification, integration, retention time correction, and peak alignment). The data matrix containing retention time, mass-to-charge ratio, and peak intensity were identified. MS and MS/MS data were used to screen for matches in the metabolite databases. The MS mass error was set at < 10 ppm and the metabolites were identified according to the secondary MS matching score. Analyzed databases included https://hmdb.ca/ and https://metlin.scripps.edu/.

The data matrix was converted using SIMCA-P software (version 14; Umetrics, Umea, Sweden) to perform a principal component analysis (PCA), a partial least squares discriminant analysis (PLS-DA), and an orthogonal partial least squares discriminant analysis (OPLS-DA). The criteria used for identifying significant differential metabolites were as follows: VIP (Variable Importance in Projection) > 1 and *P* < 0.05. Differential metabolites were analyzed qualitatively and the compound identification numbers (CIDs) were acquired using the Human Metabolome Database (https://hmdb.ca/). Differential metabolites were further annotated using the KEGG compound database (http://www.kegg.jp/kegg/compound/)^39–41^. Metabolites were annotated according to the CIDs and were mapped using the KEGG pathway database (https://www.genome.jp/kegg/pathway.html). Metabolites were classified, prominent metabolic pathways were identified, and metabolite enrichment was analyzed.

Analysis of variance, using SPSS 20.0 (SPSS Inc., Chicago, IL, USA), was conducted for biomass and LT_50_ data for the cultivars and phases. Multiple range tests were performed using least significant differences. The threshold for determining the significance of any differences was *P* < 0.05.

## Results

### Biomass and semi-lethal temperature

**Figure 2:**
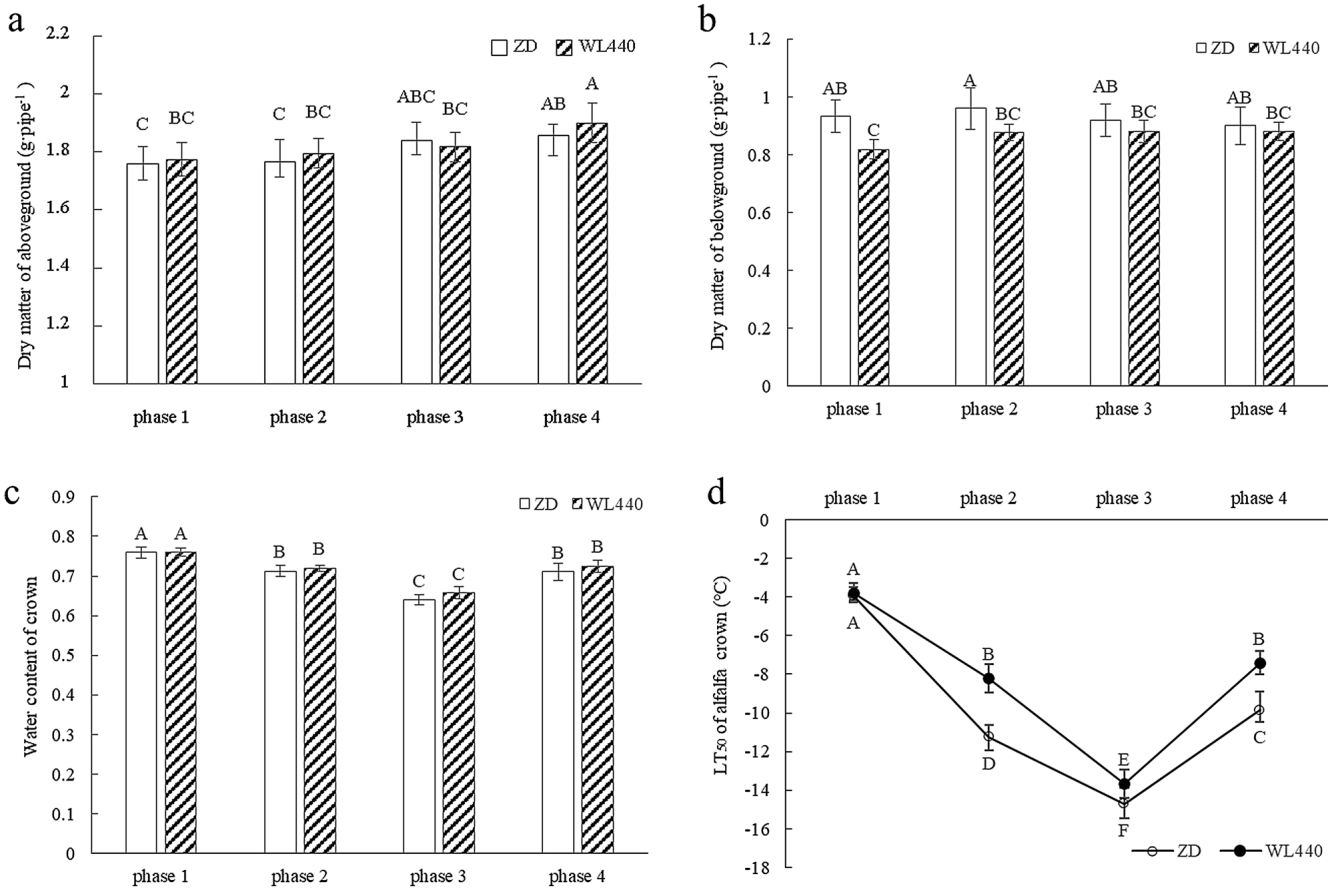
Changes of dry matter of aboveground (**a**) and belowground (**b**), water content (**c**) and semi-lethal temperature (**d**) of crown in four phases. Different capital letters represent significant differences between samples at 0.01 level.

For both cultivars, the aboveground biomass increased from phase 1 to 4, but no significant effect was detected in the within-cultivar comparisons (Fig. 2a,b). The growth rate of WL440 was greater than that of ZD in phase 4. The aboveground biomass of ZD was significantly greater than that of WL440 in phases 1 and 2. There was no significant difference in the belowground biomass of the two cultivars in any phase. From phase 1 to 4, the belowground biomass first increased and then decreased. The belowground biomass was highest in phase 2 for ZD and in phase 3 for WL440. The crown water content decreased from phase 1 to 3 and then was stable until phase 4. The crown water content did not differ significantly between the two cultivars, but there were significant differences among the four phases (Fig. 2c). There was no significant difference in the semi-lethal temperature between the two cultivars in phase 1, but there were significant differences from phase 2 to 4 (Fig. 2d). The difference in the semi-lethal temperature between the two cultivars was greatest in phase 2, after which it decreased in phase 3 and then increased in phase 4.

### Separation of metabolites

**Figure 3:**
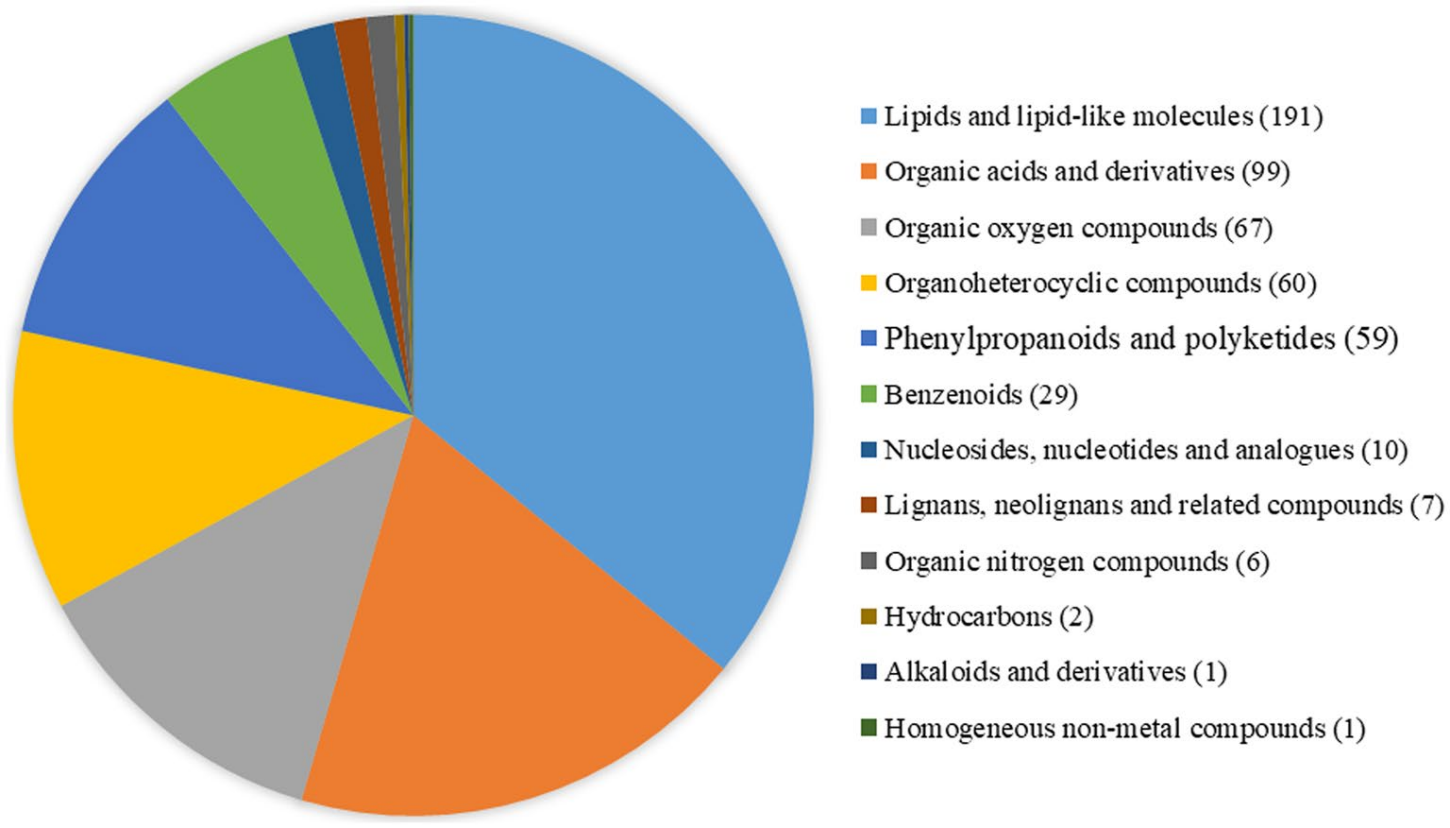
Classification of all identified metabolites at the superclass level.

A total of 532 metabolites were identified on the basis of widely targeted metabolic analyses (Table S2). These metabolites were divided into 12 superclasses (Fig. 3) and 69 classes (Table S2), including lipids and lipid-like molecules (191), organic acids and derivatives (99), organic oxygen compounds (67), organic heterocyclic compounds (60), phenylpropanoids and polyketides (59), benzenoids (29), nucleosides, nucleotides, and analogs (10), lignans, neolignans, and related compounds (7), organic nitrogen compounds (6), hydrocarbons (2), alkaloids and derivatives (1), and homogeneous non-metal compounds (1) (Fig. 3).

### Classification of differential metabolites

**Figure 4:**
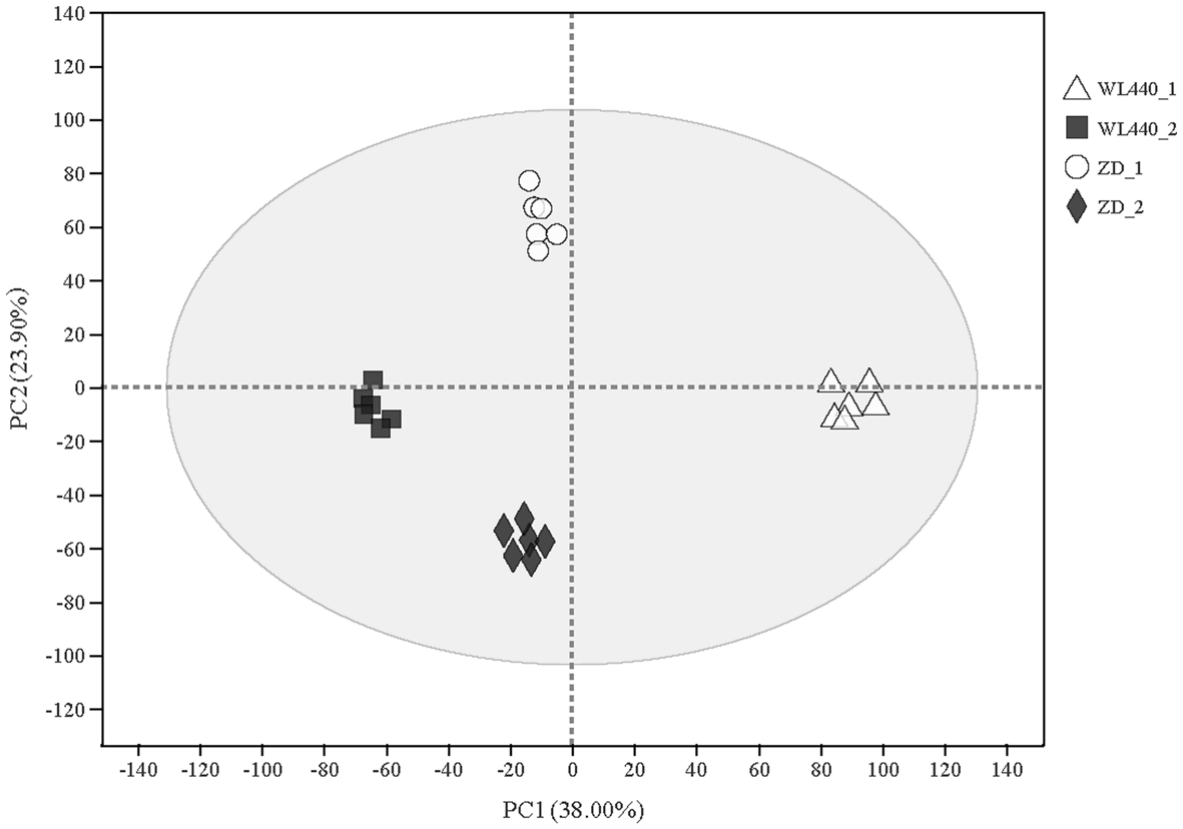
Principal component analysis (PCA) between different samples. The ellipse represents the 95% confidence interval.

PCA data (Fig. 4) show that the differential metabolites were well separated between phases and cultivars and the variation within groups was small. Using VIP > 1.0 and *P* < 0.05 as criteria, 367 differential metabolites were identified (Table S3) and classified as follows: lipids and lipid-like molecules (133), organic acids and derivatives (75), organic oxygen compounds (44), phenylpropanoids and polyketides (42), organic heterocyclic compounds (39), benzenoids (16), nucleosides, nucleotides, and analogs (7), lignans, neolignans, and related compounds (6), organic nitrogen compounds (2), alkaloids and derivatives (1), homogeneous non-metal compounds (1), and hydrocarbon (1).

**Figure 5:**
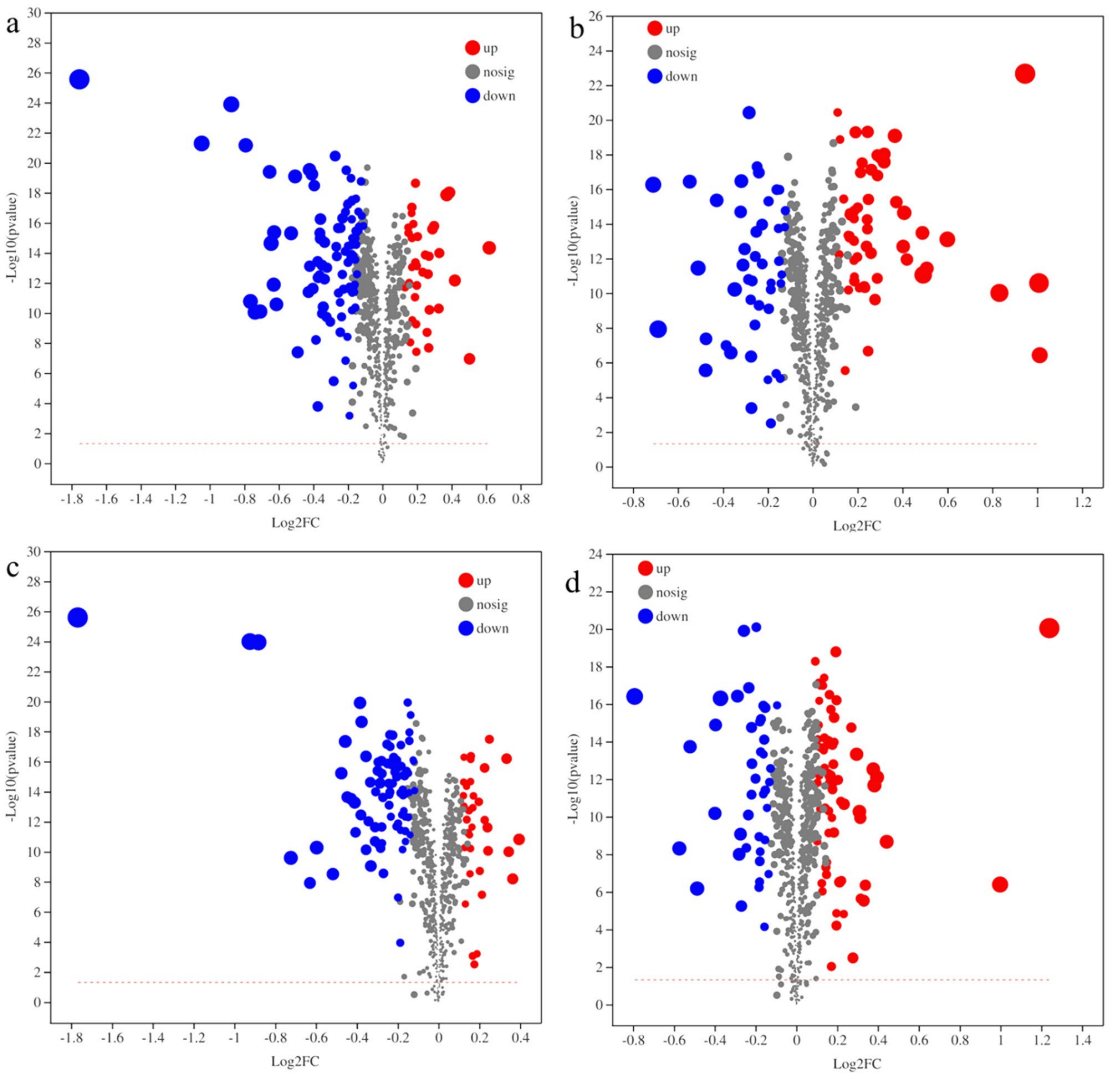
Volcano maps of differential metabolites in four pairwise comparisons: (**a**) WL440_2 vs WL440_1; (**b**) ZD_2 vs ZD_1; (**c**) ZD_1 vs WL440_1; (**d**) ZD_2 vs WL440_2. *Note*: Volcano map is used to show the relative content difference of metabolites between the two groups of samples and the statistical significance of the difference. Each point in the volcano map represents a metabolite, where red points represent up-regulated differential metabolites, gray points for non-significantly different metabolites, and green points for down-regulated differential metabolites. The abscissa represents the logarithmic value (log_2_ FC) of the multiple difference of the relative content of a metabolite in the two groups of samples. The larger the absolute value of the abscissa, the greater the relative content difference of the metabolite between the two groups of samples. The ordinate represents the VIP value, and the larger the ordinate value, the more significant difference is and the more reliable the differential metabolites obtained by screening.

Volcano maps of the differential metabolites in different pairwise comparisons are presented in Fig. 5. There were 27 up-regulated and 80 down-regulated differential metabolites in the WL440_2 vs WL440_1 comparison, whereas there were 42 up-regulated and 32 down-regulated differential metabolites in the ZD_2 vs ZD_1 comparison. The within-cultivar comparisons revealed that in WL440, the number of down-regulated metabolites increased when the temperature increased. Additionally, 25 up-regulated and 66 down-regulated differential metabolites were identified in the ZD_1 vs WL440_1 comparison, whereas 54 up-regulated and 27 down-regulated differential metabolites were identified in the ZD_2 vs WL440_2 comparison (Table 1). Specific details regarding the differential metabolites identified in the four comparison groups (e.g., classifications and fold changes) are provided in Table S4.

**Table 1:** Numbers of differential metabolites.

	WL440_2 vs WL440_1	ZD_2 vs ZD_1	ZD_1 vs WL440_1	ZD_2 vs WL440_2
All	107	74	91	81
Upregulated	27	42	25	54
Downregulated	80	32	66	27
Annotated in KEGG	23	14	19	15

**Figure 6:**
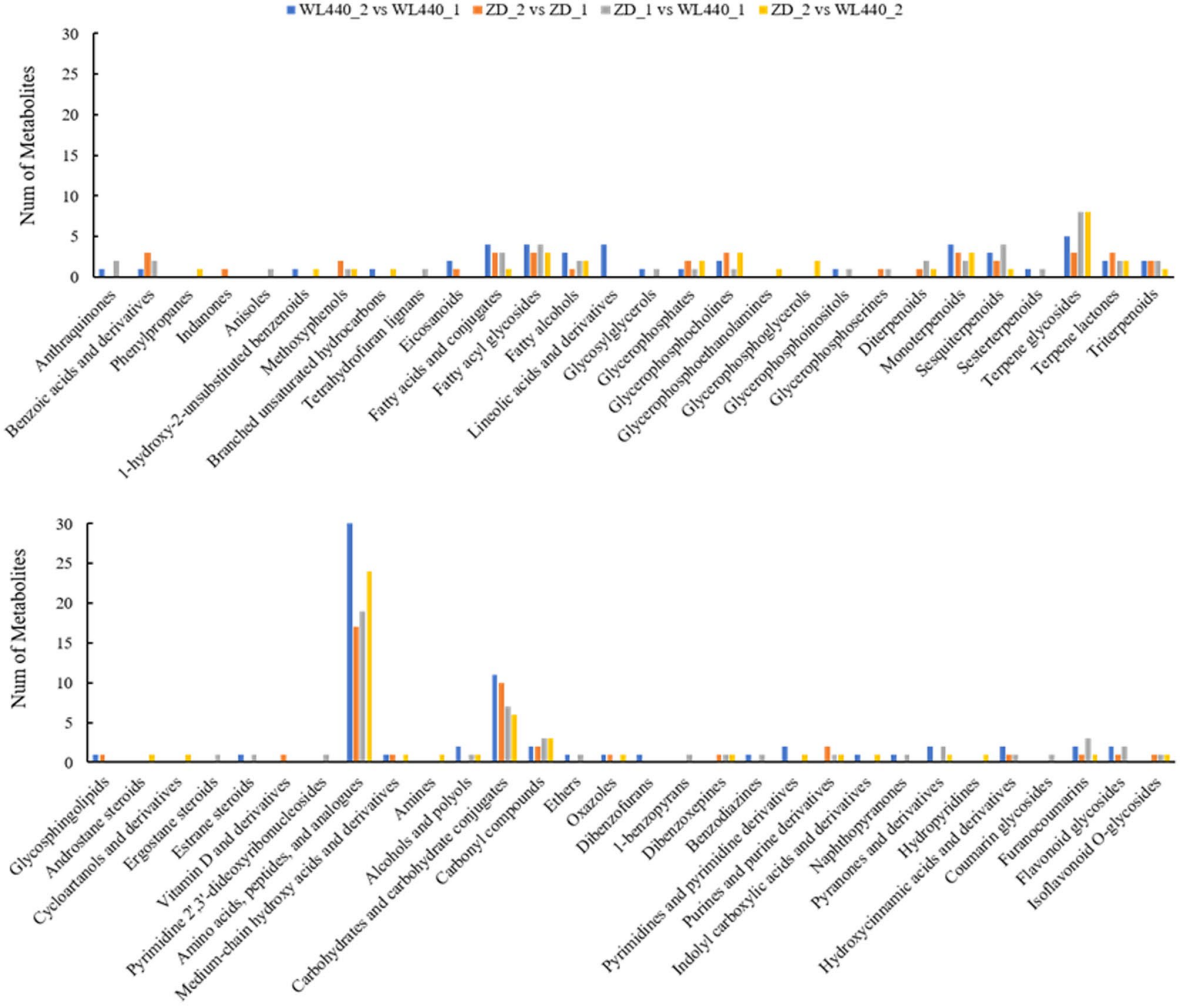
Classification of differential metabolites in four pairwise comparisons. *Note*: The abscissa represents the names of the 58 subclasses level of differential metabolites in the four pairwise comparisons.

The differing metabolites in the four pairwise comparisons (Fig. 6) were divided into 33 classes and 58 subclasses (details are provided in Table S5). Lipids and lipid-like molecules, organic acids and derivatives, and organic oxygen compounds were the most active metabolites according to the proportions of all classes of differential metabolites. At the subclass level, the differential metabolites were primarily organooxygen compounds, prenol lipids, fatty acyls, carboxylic acids and derivatives, and glycerophospholipids. Carboxylic acids and derivatives, organooxygen compounds, and prenol lipids were the main differential metabolites that were up-regulated in the WL440_2 vs WL440_1, ZD_2 vs ZD_1, and ZD_2 vs WL440_2 comparisons, whereas carboxylic acids and derivatives, prenol lipids, and glycerophospholipids were the main up-regulated differential metabolites in the ZD_1 vs WL440_1 comparison. Carboxylic acids and derivatives and prenol lipids were identified as down-regulated differential metabolites in all pairwise comparisons. The other identified down-regulated differential metabolites included fatty acyls in the WL440_2 vs WL440_1 and ZD_2 vs ZD_1 comparisons, glycerophospholipids in the ZD_2 vs ZD_1 and ZD_2 vs WL440_2 comparisons, and organooxygen compounds in the ZD_1 vs WL440_1 and ZD_2 vs WL440_2 comparisons.

### Differential metabolite analysis and enrichment

**Figure 7:**
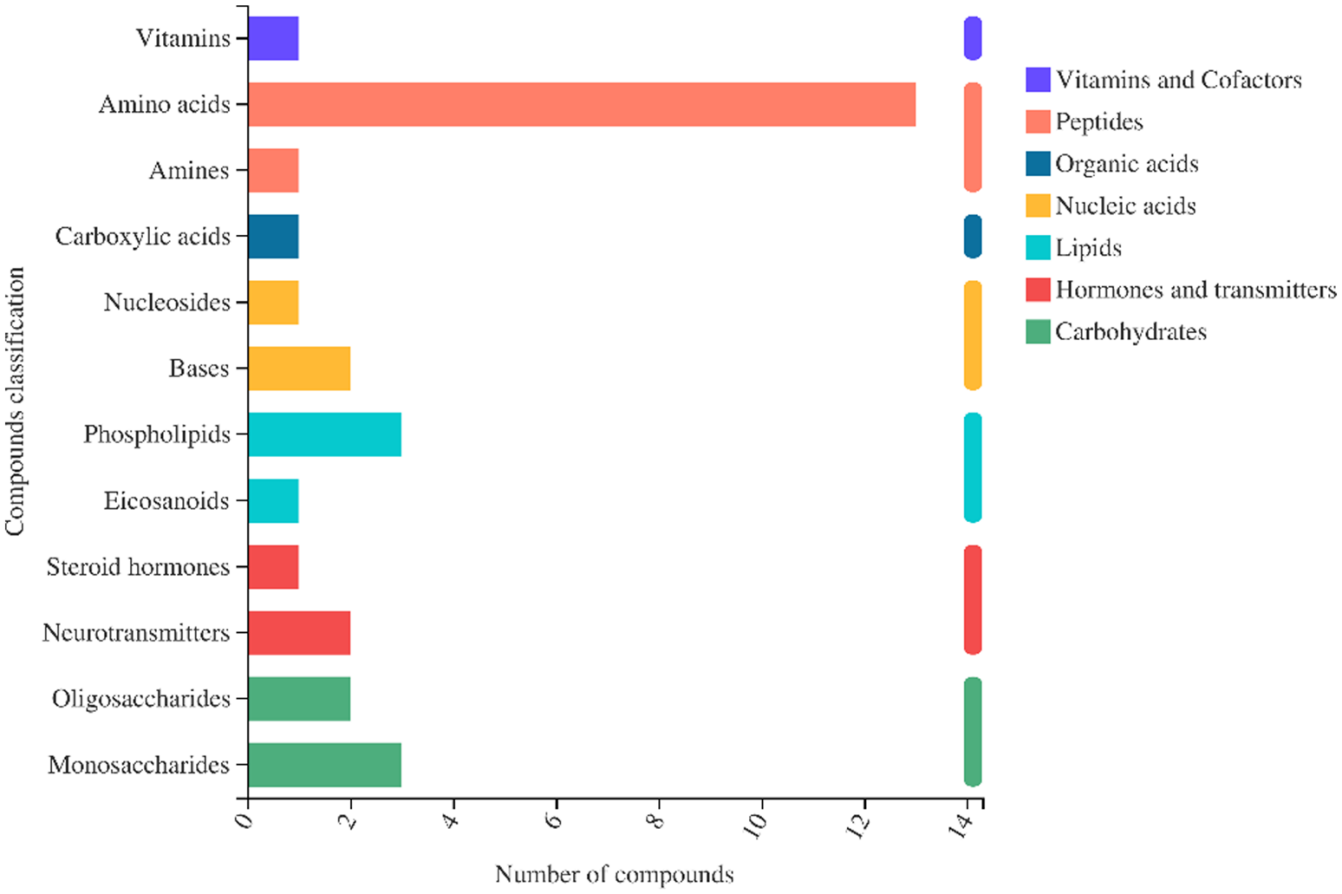
Classification of differential metabolites annotated in KEGG database.

**Figure 8:**
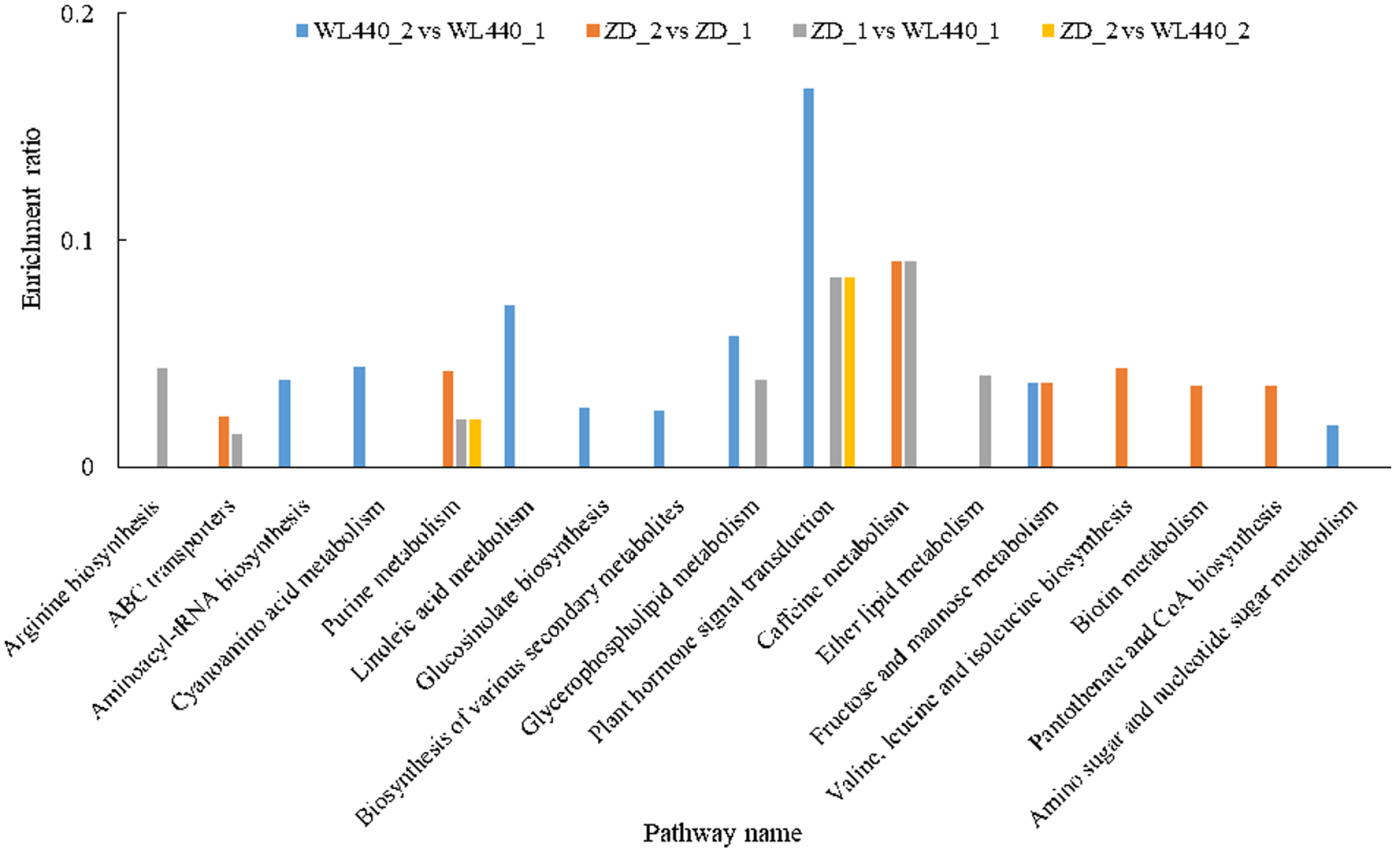
Enrichment ratios of 17 significantly (*P* < 0.05) enriched pathways.

Metabolites differed in the two cultivars differing in fall dormancy (and cold hardiness). Their metabolites and CID numbers were annotated according to the KEGG database (Fig. 7). These metabolites were divided into seven groups: peptides (14), carbohydrates (5), lipids (4), nucleic acids (3), hormones and transmitters (3), vitamins and cofactors (1), and organic acids (1). According to the KEGG pathway database, 17 major pathways were significantly enriched (*P* < 0.05) among the differential metabolites (Fig. 8). The pathways that were identified as enriched, but not significantly are listed in Table S6. At the subclass level, the metabolites associated with the 17 major pathways included amino acids, peptides and analogs, carbohydrates and carbohydrate conjugates, and glycerophosphocholines (Table 2).

**Table 2:** Changes of the differential metabolites in four pairwise comparisons that involved in 17 pathways identified as being significantly enriched in the KEGG analysis. “↓” represents a metabolite was downregulated in two comparison groups, while “↑” represents a upregulation. “—” indicates that the metabolite or a significant difference was not detected.

Metabolites	WL440_2 vs WL440_1	ZD_2 vsZD_1	ZD_1 vs WL440_1	ZD_2 vs WL440_2
**Amines**				
Porphobilinogen	—	—	—	↑
**Amino acids, peptides, and analogues**				
N, N-Dihydroxy-L-tyrosine	↓	—	↓	↑
Argininosuccinic acid	↓	—	↓	—
L-Methionine	↓	—	↓	—
Bisnorbiotin	—	↑	—	↑
Ergothioneine	—	—	—	↓
L-Valine	—	↑	—	—
**Carbohydrates and carbohydrate conjugates**				
N-Acetylmuramate	↓	—	↓	—
Stachyose	↑	↑	↓	—
L-Fucose	—	↑	—	—
Tyramine glucuronide	—	—	—	↓
**Carbonyl compounds**				
Pseudooxynicotine	↓	↑	↓	↑
**Glycerophosphates**				
Glycerophosphate	↓	—	↓	—
**Glycerophosphocholines**				
Glycerophosphocholine	↓	—	↓	—
Lysophosphatidylcholine (16:0)	—	↓	—	↓
LysoPC (15:0)	—	↓	—	↓
**Glycerophosphoethanolamines**				
Phosphatidyl ethanolamine (18:1)	—	↓	—	↓
**Indolyl carboxylic acids and derivatives**				
L-Tryptophan	—	—	—	↓
**Nucleotide metabolism**				
Hypoxanthine	—	↑	—	↑
**Purines and purine derivatives**				
Xanthine	↑	↑	↓	—
**Pyrimidines and pyrimidine derivatives**				
Cytosine	—	—	—	↓
**Sesquiterpenoids**				
Abscisic acid	↓	—	↓	↓
**Terpene glycosides**				
Geniposidic Acid	↓	—	↓	—
**Others**				
Xanthosine	↑	↑	↓	—
Inosine	—	↑	—	↑

In conclusion, carbohydrates, amino acids and lipids are the important differential metabolites responsible for the difference in cold resistance between ZD and WL440, and these metabolites are of great significance for breeding new cold-resistant alfalfa cultivars.

## Discussion

Although there is no consensus regarding the relationship between the plant tissue water content and cold hardiness, most researchers believe that cold hardiness is negatively associated with increasing water content due to lower water content can decreased osmotic pressure^5,7,8,42^. In this study, an analysis of acclimation revealed that the freezing tolerance of alfalfa was highest in phase 3 because of its decreased water content and freezing conditions (Fig. 2c,d). Additionally, cellular dehydration reportedly decreases metabolic activity, growth, and energy consumption^5^. De-acclimation and regenerative growth are related to tissue rehydration. Compared with tissues with low water contents, tissues with high water contents are more susceptible to mechanical damage caused by extracellular freezing and have a higher ice propagation rate^23^.

Alfalfa survival during a cold winter can be attributed to several causes^13,22,30^: (1) decreases in the temperature and photoperiod induce acclimation in alfalfa; (2) a continuously decreasing temperature causes alfalfa to almost stop growing, acquire peak cold hardiness, and survive the midwinter; (3) an increase in the temperature in early spring decreases the cold hardiness of alfalfa and induces regeneration. Moreover, temperature fluctuations may induce re-acclimation and de-acclimation^1,22^. Plants have evolved various strategies to minimize cellular damage in response to low-temperature stress. Examples include osmotic regulation, synthesis of soluble proteins, changes in cell membrane components, and hormonal regulation^1^.

In the current study, there was no significant difference in the cold hardiness of ZD and WL440 in phase 1, but a significant difference was detected in phase 2, possibly due to the differential metabolism of soluble sugars and amino acids^13,43^. Soluble sugars are sensitive to temperature changes. An earlier study by Bertrand^10^ demonstrated that the soluble sugar content in the root-crown region of alfalfa increases several-fold after only 8 h of a low-temperature treatment. Similarly, several recent studies have confirmed the existence of photoperiod-measuring mechanisms in the plant metabolic network that coordinates seasonal developmental programs to mitigate damages from less predictable stresses^3,4,44^. Liu^4^ reported that in Arabidopsis, changes in the cellular starch and sucrose contents can regulate the expression of photoperiod-related genes, thereby maintaining the diurnal energy level, which is crucial for plants to adapt to the relatively long and cold nights in winter. Amino acids serve as a nitrogen source for plants, but they are also important for adaptations to low-temperature stress^45,46^. Amino acids accumulate in the alfalfa root-crown region at low temperatures and specific amino acids can delay protein degradation and maintain the structural stability of the plasma membrane^47–50^.

In phase 3, there was a minor difference between the two cultivars, suggesting that sufficient cold acclimation will decrease the differences in the sensitivity to low temperatures among cultivars. Research regarding climate changes due to global warming has generated evidence that temperature fluctuations increase the risk of overwintering failure compared with the effects of an exposure to consistent low temperatures^51,52^.

Temperature increases result in changes in a series of plant metabolic pathways, which lead to the rapid loss of cold hardiness. In the current study, fucose content increased after de-acclimation. This is contrary to earlier findings^13^. As a signaling molecule and protective agent, fucose regulates plant growth and development and the mechanism mediating responses to environmental stimuli. In addition to protecting proteins and cell membranes at low temperatures, fucose also co-regulates the plant heat stress response mechanism with hormones when temperature increases^53–55^. The composition and fluidity of the plasma membrane are key factors influencing plant sensitivity to low temperatures. At low temperatures, the plasma membrane transforms from a relatively fluid structure to a solid gel, in which lipids are tightly packed and highly ordered. The solid gel membrane is highly permeable and is easily ruptured^54^. The decrease in glycerophospholipid metabolic activity during de-acclimation is believed to be related to the loss of cold hardiness. Glycerophosphate lipids, which are phospholipids comprising a phosphate and two fatty acids or fatty alcohols, are the main plasma membrane components. The comparison of different phases in this study revealed decreases in glycerophosphate, glycerophosphocholine, lysophosphatidylcholine (16:0), and lysoPC (15:0) content during de-acclimation. The decrease in unsaturated fatty acid content accelerated the loss of cold hardiness. Unsaturated fatty acids are useful for maintaining plasma membrane fluidity and cellular functions under low-temperature stress conditions, likely because of their cis-double bonds^13,15,54^. Miki^56^ conducted a proteomics-based analysis of Arabidopsis, which confirmed that changes in amino acid contents in plants are closely related to temperature. During acclimation and de-acclimation, hundreds of proteins are modified, among which transporters are the most reduced. On the basis of an analysis of the circadian clock and energy, Bonnot^44^ reported that in response to temperature limitation, organisms selectively translate mRNA to respond to environmental changes. According to a cluster analysis, most of the changes to proteins during acclimation and de-acclimation were reversible. Additionally, the changes during de-acclimation were faster than those during acclimation. In phase 4, tyrosine, succinic acid, and methionine decreased significantly in WL440, but were not significantly changed in ZD, this may help explain the observed differences between these cultivars. These findings were in accordance with the results of a previous study^57^, in which functional amino acids were observed to protect cells from cold damage.

The metabolic pathways that differed between ZD_2 and WL440_2 were mainly related to purine metabolism and plant hormone signal transduction (Fig. 8). Abscisic acid (ABA) is a sesquiterpenoid hormone that affects plant growth and development, while also regulating plant responses to adverse environmental conditions. Furthermore, ABA-dependent signaling is one of the pathways responsive to low-temperature stress^58–60^. An exposure to low temperatures leads to an increase in ABA content. The accumulated ABA binds to receptors, which then interact with PP2C (type 2C protein phosphatase) to inhibit the binding of PP2C to SnRK2 (SNFI-related protein kinase 2). As a result, SnRK2 transcription factors are phosphorylated, which can activate the expression of ABA-responsive genes and increase cold hardiness^61,62^.

The mechanisms underlying alfalfa plant responses to cold conditions are primarily associated with changes in amino acids, lipid molecules, carbohydrates, and their related metabolic pathways. At different stages, various metabolites contribute to alfalfa cold hardiness. For example, alfalfa adapts to low-temperature environments via an increase in the abundance of amino acids (e.g., aspartic acid), fatty acids (e.g., arachidonic acid and norlinolenic acid), glycerophospholipids (e.g., lysophosphatidylcholine and phosphatidyl ethanolamine), and carbohydrates (e.g., glucose, maltose, and raffinose) in the root-crown region during phase 2^13^. However, in phase 3, the contents of most of these metabolites are no longer increased, and may be decreased (with the exception of raffinose). Cystathionine and maltotriose content increase in phase 3^13^. Alfalfa appears to cope with temperature increases mainly through decreases in various amino acid and glycerophospholipid contents, including tyrosine, succinic acid, methionine, glycerophosphate, glycerophosphocholine, lysophosphatidylcholine (16:0), and lysoPC (15:0) (Table 2). These observations, combined with the results of previous studies by Xu^1,13^, highlight the importance of amino acids and glycerophospholipids in both cold acclimation and de-acclimation. Their contents increase during cold acclimation and decrease during de-acclimation. Soluble sugars are mainly involved in increasing the cold hardiness of alfalfa as part of the cold acclimation process.

## Conclusions

Differences in the cold hardiness of two alfalfa cultivars gradually decreased in the continuous cold acclimation period, but increased during de-acclimation. These results indicate that the rapid decrease in the cold hardiness of plants in early spring is an important factor related to the inhibition of alfalfa regrowth. Results of the metabolomic analysis showed that ZD had better cold resistance than WL440 during both cold acclimation and de-acclimation, which was related to the change rate of amino acid and glycerophospholipids content in the two periods. In contrast, the primary function of soluble sugars is to improve the cold hardiness of alfalfa during the cold acclimation period. Data provided herein proves that soluble sugars, amino acids and lipids are important metabolites affecting the cold resistance of alfalfa, and it is further clarified that unsaturated fatty acids are conducive to improving the cold resistance. Furthermore, the study results provide a reference for breeding new cold-resistant alfalfa cultivars.

## Supplementary Information

Supplementary Information 1.

Supplementary Information 2.

Supplementary Information 3.

Supplementary Information 4.

Supplementary Information 5.

Supplementary Information 6.

Supplementary Information 7.

## Data Availability

All data generated or analysed during this study are included in this published article [and its supplementary information files].

## References

[1] Xu HY, Tong ZY, He F, Li XL (2020). Response of alfalfa (*Medicago sativa* L.) to abrupt chilling as reflected by changes in freezing tolerance and soluble sugars. Agronomy.

[2] Li Z, Wan L, Li S, Li X, He F, Tong Z (2021). Plastic response of *Medicago sativa* L. root system traits and cold resistance to simulated rainfall events. PeerJ.

[3] Kidokoro S, Hayashi K, Haraguchi H, Ishikawa T, Soma F, Konoura I, Toda S, Mizoi J, Suzuki T, Shinozaki K, Yamaguchi-Shinozaki K (2021). Posttranslational regulation of multiple clock-related transcription factors triggers cold-inducible gene expression in *Arabidopsis*. Proc. Natl. Acad. Sci. U S A.

[4] Liu W, Feke A, Leung CC, Tarte DA, Yuan W, Vanderwall M, Sager G, Wu X, Schear A, Clark DA, Thines BC, Gendron JM (2021). A metabolic daylength measurement system mediates winter photoperiodism in plants. Dev. Cell.

[5] Kalberer SR, Wisniewski M, Arora R (2006). Deacclimation and reacclimation of cold-hardy plants: Current understanding and emerging concepts. Plant Sci..

[6] Webster DE, Ebdon JS (2005). Effects of nitrogen and potassium fertilization on perennial ryegrass cold tolerance during deacclimation in late winter and early spring. HortScience.

[7] Panjtandoust M, Wolyn DJ (2016). Freezing tolerance attributes during spring deacclimation for three asparagus cultivars with varying adaptation to Southern Ontario. J. Agron. Crop Sci..

[8] Panjtandoust M, Wolyn DJ, Navabi A (2016). Asparagus cultivars with varying adaptation to southern Ontario differ for induction of freezing tolerance in the fall. Can. J. Plant Sci..

[9] Ashworth EN (1992). Formation and spread of ice in plant tissues. Hortic. Rev..

[10] Bertrand A, Bipfubusa M, Claessens A, Rocher S, Castonguay Y (2017). Effect of photoperiod prior to cold acclimation on freezing tolerance and carbohydrate metabolism in alfalfa (*Medicago sativa* L.). Plant Sci..

[11] Cunningham SM, Nadeau P, Castonguay Y, Laberge S, Volenec JJ (2003). Raffinose and stachyose accumulation, galactinol synthase expression, and winter injury of contrasting alfalfa germplasms. Crop Sci..

[12] Haagenson DM, Cunningham SM, Joern BC, Volenec JJ (2003). Autumn defoliation effects on alfalfa winter survival, root physiology, and gene expression. Crop Sci..

[13] Xu HY, Li ZY, Tong ZY, He F, Li XL (2020). Metabolomic analyses reveal substances that contribute to the increased freezing tolerance of alfalfa (*Medicago sativa* L.) after continuous water deficit. BMC Plant.

[14] Zeng Y, Yu J, Cang J, Liu L, Mu Y, Wang J, Zhang D (2014). Detection of sugar accumulation and expression levels of correlative key enzymes in winter wheat (*Triticum aestivum*) at low temperatures. Biosci. Biotechnol. Biochem..

[15] Uemura M, Joseph RA, Steponkus PL (1995). Cold acclimation of *Arabidopsis thaliana* (effect on plasma membrane lipid composition and freeze-induced lesions). Plant Physiol..

[16] Steponkus PL (1984). Role of the plasma membrane in freezing injury and cold acclimation. Annu. Rev. Plant Physiol..

[17] Bohn M, Luthje S, Sperling P, Heinz E, Dorffling K (2007). Plasma membrane lipid alterations induced by cold acclimation and abscisic acid treatment of winter wheat seedlings differing in frost resistance. J. Plant Physiol..

[18] Sung DY, Kaplan F, Lee KJ, Guy CL (2003). Acquired tolerance to temperature extremes. Trends Plant Sci..

[19] Cyril J, Powell GL, Duncan RR, Baird WV (2002). Changes in membrane polar lipid fatty acids of seashore paspalum in response to low temperature exposure. Crop Sci..

[20] Augspurger CK (2009). Spring 2007 warmth and frost: phenology, damage and refoliation in a temperate deciduous forest. Funct. Ecol..

[21] Browse J, Lange BM (2004). Counting the cost ofa cold-blooded life: Metabolomics ofcold acclimation. Proc. Natl. Acad. Sci. U S A.

[22] Kalberer SR, Leyva-Estrada N, Krebs SL, Arora R (2007). Frost dehardening and rehardening of floral buds of deciduous azaleas are influenced by genotypic biogeography. Environ. Exp. Bot..

[23] Ogren E (1996). Premature dehardening in vaccinium myrtillus during a mild winter: A cause for winter dieback?. Funct. Ecol..

[24] Gusta LV, Fowler DB (1976). Effects of temperature on dehardening and rehardening of winter cereals. Can. J. Plant Sci..

[25] Gusta LV, Fowler DB (1976). Dehardening and rehardening of spring-collected winter wheats and a winter rye. Can. J. Plant Sci..

[26] Ishikawa M, Sakai A (1981). Freezing avoidance mechanisms by supercooling in some *Rhododendron* flower buds with reference to water relations. Plant Cell Physiol..

[27] Andrews PK, Proebsting EL (1987). Effects of temperature on the deep supercooling characteristics of dormant and deacclimating sweet. J. Am. Soc. Hortic. Sci..

[28] Ogren E (1997). Relationship between temperature, respiratory loss of sugar and premature dehardening in dormant Scots pine seedlings. Tree Physiol..

[29] Rapacz M (2000). Frost De-acclimation of Barley (*Hordeum vulgare* L.) and Meadow Fescue (*Festuca pratensis* Huds.). Relationship between soluble carbohydrate content and resistance to frost and the fungal PathogenBipolaris sorokiniana (Sacc.) Shoem. Ann. Bot..

[30] Trischuk RG, Schilling BS, Low NH, Gray GR, Gusta LV (2014). Cold acclimation, de-acclimation and re-acclimation of spring canola, winter canola and winter wheat: The role of carbohydrates, cold-induced stress proteins and vernalization. Environ. Exp. Bot..

[31] Tronsmo AM, Kobro G, Morgenlie S, Flengsrud R (1993). Carbohydrate content and glycosidase activities following cold hardening in two grass species. Physiol. Plant..

[32] Sasaki H, Ichimura K, Oda M (1996). Changes in sugar content during cold acclimation and deacclimation of cabbage seedlings. Ann. Bot..

[33] Flinn CL, Ashworth EN (1995). The relationship between carbohydrates and flower bud hardiness among three *Forsythia taxa*. J. Am. Soc. Hortic. Sci..

[34] Iivonen S, Saranpää P, Sutinen ML, Vapaavuori E (2004). Effects of temperature and nutrient availability on plasma membrane lipid composition in Scots pine roots during growth initiation. Tree Physiol..

[35] Yoshida S (1986). Reverse changes in plasma membrane properties upon deacclimation of mulberry trees (*Morus bombysis *Koidz.). Plant Cell Physiol..

[36] Fahy E, Subramaniam S, Brown HA, Glass CK, Merrill AH, Murphy RC, Raetz CR, Russell DW, Seyama Y, Shaw W, Shimizu T, Spener F, van Meer G, VanNieuwenhze MS, White SH, Witztum JL, Dennis EA (2005). A comprehensive classification system for lipids. J. Lipid Res..

[37] Welti R, Li W, Li M, Sang Y, Biesiada H, Zhou HE, Rajashekar CB, Williams TD, Wang X (2002). Profiling membrane lipids in plant stress responses. Role of phospholipase D alpha in freezing-induced lipid changes in *Arabidopsis*. J. Biol. Chem..

[38] Anower MR, Fennell A, Boe A, Mott IW, Peel MD, Wu Y (2016). Physiological and molecular characterisation of lucerne (*Medicago sativa* L.) germplasm with improved seedling freezing tolerance. Crop Pasture Sci..

[39] Kanehisa M, Goto S (2000). KEGG: Kyoto encyclopedia of genes and genomes. Nucleic Acids Res..

[40] Kanehisa M (2019). Toward understanding the origin and evolution of cellular organisms. Protein Sci..

[41] Kanehisa M, Furumichi M, Sato Y, Ishiguro-Watanabe M, Tanabe M (2021). KEGG: Integrating viruses and cellular organisms. Nucleic Acids Res..

[42] Landry EJ, Wolyn DJ (2011). Cold acclimation attributes of two asparagus cultivars with varying patterns of fern senescence. J. Am. Soc. Hortic. Sci..

[43] Reyes-Diaz M, Ulloa N, Zuniga-Feest A, Gutierrez A, Gidekel M, Alberdi M, Corcuera LJ, Bravo LA (2006). Arabidopsis thaliana avoids freezing by supercooling. J. Exp. Bot..

[44] Bonnot T, Nagel DH (2021). Time of the day prioritizes the pool of translating mRNAs in response to heat stress. Plant Cell.

[45] Zhao M, Ren Y, Wei W, Yang J, Zhong Q, Li Z (2021). Metabolite analysis of jerusalem artichoke (*Helianthus tuberosus* L.) seedlings in response to polyethylene glycol-simulated drought stress. Int. J. Mol. Sci..

[46] Yang Y, Jia Z, Chen F, Sang Z, Ma L (2015). Comparative analysis of natural cold acclimation and deacclimation of two magnolia species with different winter hardiness. Acta Physiol. Plant..

[47] Hoffman L, DaCosta M, Ebdon JS (2014). Examination of cold deacclimation sensitivity of annual bluegrass and creeping bentgrass. Crop Sci..

[48] Dhont C, Castonguay Y, Nadeau P, Belanger G, Drapeau R, Laberge S, Avice JC, Chalifour FP (2006). Nitrogen reserves, spring regrowth and winter survival of field-grown alfalfa (*Medicago sativa*) defoliated in the autumn. Ann. Bot..

[49] Khan N, Bano A, Rahman MA, Rathinasabapathi B, Babar MA (2019). UPLC-HRMS-based untargeted metabolic profiling reveals changes in chickpea (*Cicer arietinum*) metabolome following long-term drought stress. Plant Cell Environ..

[50] Jia H, Wang L, Li J, Sun P, Lu M, Hu J (2020). Comparative metabolomics analysis reveals different metabolic responses to drought in tolerant and susceptible poplar species. Physiol. Plant.

[51] Carol KA (2013). Reconstructing patterns of temperature, phenology, and frost damage over 124 years. Ecology.

[52] Ji HT, Xiao LJ, Xia YM, Song H, Liu B, Tang L, Cao WX, Zhu Y, Liu LL (2017). Effects of jointing and booting low temperature stresses on grain yield and yield components in wheat. Agric. For. Meteorol..

[53] Suzuki N, Bajad S, Shuman J, Shulaev V, Mittler R (2008). The transcriptional co-activator MBF1c is a key regulator of thermotolerance in *Arabidopsis thaliana*. J. Biol. Chem..

[54] Arbona V, Manzi M, Ollas C, Gomez-Cadenas A (2013). Metabolomics as a tool to investigate abiotic stress tolerance in plants. Int. J. Mol. Sci..

[55] Lastdrager J, Hanson J, Smeekens S (2014). Sugar signals and the control of plant growth and development. J. Exp. Bot..

[56] Miki Y, Takahashi D, Kawamura Y, Uemura M (2019). Temporal proteomics of *Arabidopsis* plasma membrane during cold- and de-acclimation. J. Proteomics.

[57] Peng Y, Arora R, Li G, Wang X, Fessehaie A (2008). Rhododendron catawbiense plasma membrane intrinsic proteins are aquaporins, and their over-expression compromises constitutive freezing tolerance and cold acclimation ability of transgenic *Arabidopsis* plants. Plant Cell Environ..

[58] Wang X, Guo C, Peng J, Li C, Wan F, Zhang S, Zhou Y, Yan Y, Qi L, Sun K, Yang S, Gong Z, Li J (2019). ABRE-BINDING FACTORS play a role in the feedback regulation of ABA signaling by mediating rapid ABA induction of ABA co-receptor genes. New Phytol..

[59] Huang X, Hou L, Meng J, You H, Li Z, Gong Z, Yang S, Shi Y (2018). The antagonistic action of abscisic acid and cytokinin signaling mediates drought stress response in *Arabidopsis*. Mol. Plant.

[60] Li H, Ye K, Shi Y, Cheng J, Zhang X, Yang S (2017). BZR1 positively regulates freezing tolerance via CBF-dependent and CBF-independent pathways in arabidopsis. Mol. Plant.

[61] Ma Y, Szostkiewicz I, Korte A, Moes D, Yang Y, Christmann A, Grill E (2009). Regulators of PP2C phosphatase activity function as abscisic acid sensors. Science.

[62] Sah SK, Reddy KR, Li J (2016). Abscisic acid and abiotic stress tolerance in crop plants. Front Plant Sci..

