# Supplementary material for: Metabolomic changes in crown of alfalfa (Medicago sativa L.) during de-acclimation
Source: Sci Rep. 2022 Sep 2;12:14977. doi: 10.1038/s41598-022-19388-x (PMC9440230; doi:10.1038/s41598-022-19388-x)
Supplement: Supplementary file 1 — Supplementary Information 1. [file 41598_2022_19388_MOESM1_ESM.docx]

Table S1 Mobile phase elution gradient (Table 1) and mass spectrum parameters (Table 2).

Table S2 All metabolites were identified (Sheet 1) and their classification at the class level (Sheet 2) by widely targeted in this study.

Table S3 Information on 367 differential metabolites (Sheet 1) and their classification at superclass level (Sheet 2).

Table S4 Information on differential metabolites identified in the four comparison groups (as shown in the sheet name).

Table S5 Information on the classification of differential metabolites identified in the four comparison groups at class and subclass level.

Table S6 Insignificantly enriched pathways in differential metabolites.
