# Supplementary material for: Metabolomic changes in crown of alfalfa (Medicago sativa L.) during de-acclimation
Source: Sci Rep. 2022 Sep 2;12:14977. doi: 10.1038/s41598-022-19388-x (PMC9440230; doi:10.1038/s41598-022-19388-x)
Supplement: Supplementary file 2 — Supplementary Information 2. [file 41598_2022_19388_MOESM2_ESM.docx]

Table 1 Mobile phase elution gradient

| Time (min) | Flow rate (mL/min) | A (%) | B (%) |
| --- | --- | --- | --- |
| 0 | 0.4 | 100 | 0 |
| 3.5 | 0.4 | 75.5 | 24.5 |
| 5 | 0.4 | 35 | 65 |
| 5.5 | 0.4 | 0 | 100 |
| 7.4 | 0.6 | 0 | 100 |
| 7.6 | 0.6 | 48.5 | 51.5 |
| 7.8 | 0.5 | 100 | 0 |
| 9 | 0.4 | 100 | 0 |
| 10 | 0.4 | 100 | 0 |

Table 2 Mass spectrum parameters

| Description | Parameter |
| --- | --- |
| Scan type (m/z) | 70-1050 |
| Sheath gas flow rate (arb) | 50 |
| Aux gas flow rate (arb) | 13 |
| Heater temp (℃) | 425 |
| Capillary temp (℃) | 325 |
| Spray voltage (+) (V) | 3500 |
| Spray voltage (-) (V) | -3500 |
| S-Lens RF Level | 50 |
| Normalized collision energy (eV) | 20, 40, 60 |
| Resolution (Full MS) | 60000 |
| Resolution (MS^2^) | 7500 |
